# Towards automated phenotype definition extraction using large language models

**DOI:** 10.1186/s44342-024-00023-2

**Published:** 2024-10-31

**Authors:** Ramya Tekumalla, Juan M. Banda

**Affiliations:** 1 Mercer University, Atlanta, GA USA; 2 Stanford Health Care, Stanford, CA USA; 3 Observational Health Data Sciences and Informatics, New York, NY USA

**Keywords:** ChatGPT, Electronic phenotyping, Large language models (LLMs), Evaluation

## Abstract

Electronic phenotyping involves a detailed analysis of both structured and unstructured data, employing rule-based methods, machine learning, natural language processing, and hybrid approaches. Currently, the development of accurate phenotype definitions demands extensive literature reviews and clinical experts, rendering the process time-consuming and inherently unscalable. Large language models offer a promising avenue for automating phenotype definition extraction but come with significant drawbacks, including reliability issues, the tendency to generate non-factual data (“hallucinations”), misleading results, and potential harm. To address these challenges, our study embarked on two key objectives: (1) defining a standard evaluation set to ensure large language models outputs are both useful and reliable and (2) evaluating various prompting approaches to extract phenotype definitions from large language models, assessing them with our established evaluation task. Our findings reveal promising results that still require human evaluation and validation for this task. However, enhanced phenotype extraction is possible, reducing the amount of time spent in literature review and evaluation.

## Introduction

In the era of digital healthcare, the advent of electronic health records (EHRs) and the proliferation of digital health data are catalyzing a paradigm shift in medical research and patient care. At the heart of this transformation is electronic phenotyping, a process that utilizes these vast datasets to identify and classify patient phenotypes. Phenotyping, in the context of biomedical research, refers to the process of extracting relevant health characteristics from patient data that can be correlated with specific health outcomes, diseases, or conditions. This process is critical for advancing personalized medicine, streamlining patient care, and driving forward biomedical discoveries. Electronic phenotyping harnesses both structured and unstructured data, integrating rule-based systems, machine learning techniques, natural language processing (NLP), and hybrid methodologies to analyze and categorize patient information [1].

The significance of electronic phenotyping cannot be overstated; it forms the cornerstone of efforts to tailor healthcare to individual patient needs [2], enhance the understanding of disease mechanisms[3], and facilitate the development of novel therapeutic interventions. However, the scalability of electronic phenotyping poses a formidable challenge. Currently, defining a phenotype requires exhaustive literature reviews and intensive collaboration among clinicians, domain experts, and researchers to achieve consensus on precise phenotype definitions [4]. This iterative and collaborative process is time-consuming and resource-intensive, making the current approach to phenotyping less scalable and adaptable to the fast-paced advancements in medical research and emerging health crises. As the volume of digital health data explodes and the complexity of diseases becomes more apparent, the ability to quickly and accurately define, refine, and utilize phenotypes is paramount [5]. Scalable and portable phenotyping processes can accelerate the pace of research, enable the rapid identification of patient cohorts for clinical trials, and improve the detection and treatment of diseases at an individual level [6].

Leveraging machine learning for electronic phenotyping introduces a scalable approach to processing and interpreting healthcare data, fundamentally shifting the paradigm from manual, labor-intensive methods to automated, data-driven insights [7–9]. In the beginning, both rule-based and machine learning models were utilized to identify phenotypes [10]. However, the expansion of large language models (LLMs) to include hundreds of billions of parameters has introduced new abilities like few-shot learning [11]. This development enables LLMs to achieve good performance on tasks with minimal training, using only a small number of examples [12]. Several large language models like PhenoBCBERT and PhenoGPT are accurately able to infer essential phenotypic information from the given context [13]. This rapid increase in experimentation with LLMs has created pathways for researchers to utilize LLMs for electronic phenotyping.

In this work, we propose an innovative approach to address the scalability challenge in electronic phenotyping. Our work is anchored in two main objectives: first, to define a standard evaluation task/set specifically tailored for this domain, and second, to evaluate various prompting approaches for extracting phenotype definitions from LLMs. The establishment of a standard evaluation task is crucial as it serves as a benchmark to ensure that the outputs produced by LLMs are not only useful but reliable. Following this, we explore and assess different prompting strategies to effectively extract phenotype definitions from LLMs, utilizing the evaluation task we have created. Additionally, we focus on the behavior of LLMs. This dual approach represents a significant step forward in automating the phenotype definition process, leveraging the advanced capabilities of LLMs to interpret and generate natural language. By doing so, we aim to significantly reduce the time and effort currently required to define phenotypes, thereby enhancing the scalability and efficiency of electronic phenotyping. Our exploration into the use of LLMs for phenotype definition extraction is intended to pave the way for more scalable and adaptable phenotyping processes, ultimately accelerating innovation and improving outcomes in healthcare and biomedical research.

## Data preparation

The primary objective of this work is to create an evaluation set. We identified 10 professionally created phenotypes, 5 from PheKB [14] and 5 from the OHDSI phenotype library [15]. The extractions of the phenotypes from sources like OHDSI phenotype library and HDRUK phenotype library [16] are relatively easier as the phenotypes are in a structured format. OHDSI uses the OMOP (Observational Medical Outcomes Partnership) Common Data Model (CDM), which standardizes healthcare data into a consistent format, facilitating efficient and scalable analysis across different databases. This standardization reduces the complexity and effort required to extract and analyze phenotypes, as researchers can apply the same query across multiple datasets without needing to adjust for disparate data structures However, PheKB provides a platform for developing, validating, and sharing phenotype algorithms without mandating a specific data model. This approach offers flexibility and can accommodate a wide variety of data structures but may require more effort to adapt and apply algorithms across different EHR systems and databases. Hence, manual curation was required to format the PheKB phenotype definitions. We developed an automated computer code to automatically extract and format the elements from the phenotypes to facilitate automatic evaluation. Table 1 presents the extracted elements of the 10 professionally created phenotypes. The code count in the following table refers to the frequency or occurrence of a specific code within a dataset or a set of criteria. For example, “1” indicates that the code is required or included exactly once within the criteria and “1 + ” indicates that the code can be included more than once, meaning it might appear multiple times or is required at least once but can occur multiple times in the dataset or phenotype definition. The “Logic” column defines the role that each code plays in the overall logical framework of the phenotype. It determines whether the presence of a particular condition (represented by the code) qualifies a patient for inclusion or exclusion in the phenotype group being studied or defined.

**Table 1:** Extracted elements from the phenotypes

Logic	Vocabulary	Concept code	Concept name	Code count
Inclusion	SNOMED	194823009	Acute coronary insufficiency	1 +
Inclusion	SNOMED	791000119109	Angina associated with type 2 diabetes mellitus	1
Inclusion	SNOMED	61490001	Angina, class I	1 +
Inclusion	SNOMED	41334000	Angina, class II	1
Inclusion	SNOMED	85284003	Angina, class III	1 +
Inclusion	SNOMED	89323001	Angina, class IV	1 +

## Evaluation setup

One of our objectives in this work is to evaluate prompting approaches to extract phenotype definitions from LLMs and assess them using the evaluation set created in the data preparation section. We experimented with several prompts to create a prompt which can be utilized for extracting all elements of a phenotype and finally used a prompt which brought in relatively consistent results from the LLMs. We experimented with several prompting methods like zero shot, one-shot, iterative prompting, seeding, and finally developed a prompt. The following is our final prompt used for evaluation: “Provide a computational phenotype for < INSERT_PHENOTYPE > with codes needed and their name, and logical conditions as well as how many codes are needed. In the following tabular format: Logic (inclusion or exclusion), code vocabulary, code identifier, code name, and code count.”

To evaluate the efficiency of LLMs, we considered two different scenarios. In the first scenario, we compared the definitions extracted by GPT 3.5 and GPT 4. In the second scenario, we compared the definitions extracted by GPT4 and manually curated definitions (by humans). In both the scenarios, we present the following metrics:

- Overlap of codes: This metric refers to the extent to which the codes generated by GPT models match or overlap with the codes found in the original phenotype definitions.
- Logic matching: This metric refers to the degree to which the logical structure or conditions (such as inclusion/exclusion criteria) in the model’s output align with those in the original phenotype definitions.
- Overlap of strings: This metric measures the overlap in the text or strings of words between the output of the GPT model and the original phenotype definitions.

Additionally, we measured the inconsistencies and incorrect definitions and presented them in our discussion.

## Results

We present the results for scenarios 1 and 2 in Tables 2 and 3. We calculated the average, minimum, and maximum percentage of each of the metrics (e.g., codes overlap).

**Table 2:** Comparison between GPT 3.5 vs GPT 4

Metric	Average %	Minimum %	Maximum %
Codes overlap	41.26	0.00	75.00
Logic overlap	80.00	50.00	100.00
Strings overlap	28.52	0.00	50.00

**Table 3:** Comparison between human definition vs GPT models

Model	Metric	Average %	Minimum %	Maximum %
	Codes overlap	50.94	20.00	88.89
GPT 4	Logic overlap	90.00	50.00	100.00
	Strings overlap	48.59	0.00	100.00
	Codes overlap	27.51	10.00	85.20
GPT 3.5	Logic overlap	70.20	0.00	90.00
	Strings overlap	41.28	0.00	75.12

The key findings of this scenario indicate that GPT models are better in generating precise codes over textual strings. There is a considerable variability in the models’ outputs indicating a challenge in achieving consistent results across different iterations. An interesting result here is that LLMs demonstrate solid competency while extracting the logical conditions of inclusion/exclusion of codes in phenotype definitions. These insights show that one potential reason for the low overlap in codes and strings within definitions is the great variability of code systems used in phenotype definitions found in literature and on the definitions themselves [17]. We theorize that papers and abstracts are part of the GPT model training sets, and this is reflected in the inconsistent LLM output.

A noteworthy observation is that the codes generated by GPT-4 exhibit a marginally higher reliability compared to the textual strings or concept names. The codes denominator for the codes overlap metric is the number of codes from GPT4. Furthermore, despite the overall fewer codes generated by GPT-4, a closer examination suggests that these codes may possess a higher positive predictive value (PPV) for accurately identifying the intended phenotypes. This finding suggests that while the volume of generated codes is limited, their specificity and relevance to the phenotypes are notably high, indicating that the model might be averaging out from source and could be surfacing the most popular ones. Table 4 presents the GPT hallucinations with codes. In this work, we compared the performance of GPT-3.5 and GPT-4 models in generating phenotype codes, using Biomedical Content Explorer [18] linked with PubDictionaries, ICD10, and ICD 10 CM dictionaries; with this comparison, we show the biggest weakness of these LLM model as it is highly inaccurate and full of hallucinations. We discovered that hallucinations were notably present in both models, with GPT-3.5 showing a higher tendency towards these inaccuracies compared to GPT-4. These observations emphasize the imperative for cautious use and meticulous verification of data produced by LLMs, especially for phenotypes less documented in scientific literature. The pattern observed suggests a direct relationship between the scarcity of literature on specific phenotypes and the models’ propensity to generate non-existent codes, pointing to a crucial area for enhancement in the training methodologies of these models.

**Table 4:** Comparisons of GPT hallucinations when producing codes

Model	Average %	Minimum %	Maximum %
GPT 3.5	38	0	83
GPT 4	32	0	69

Additionally, we performed a detailed evaluation of the capabilities of GPT-3.5 and GPT-4 models in accurately extracting phenotype definitions, a crucial step toward their integration into medical informatics. This entails an extensive series of evaluations comparing these large language models (LLMs) against human-generated definitions to assess various aspects: the accuracy of code mapping, the consistency of code names, the logical structuring of definitions, and the degree of overlap in the codes identified. In our experiment, we compared human-generated definitions of phenotypes against those produced by GPT-4, focusing on the process of code mapping to align disparate coding systems into a unified framework. Table 5 presents the results of our evaluations.

**Table 5:** Code mapping evaluation of GPT models, comparing codes as extracted vs mapped by the LLM

Model	Metric	Average %	Minimum %	Maximum %
GPT 4	Extracted codes overlap	50.94	20.00	89.00
	Mapped codes overlap	72.89	28.98	97.00
GPT 3.5	Extracted codes overlap	27.51	10.00	85.20
	Mapped codes overlap	58.15	19.87	62.20

## Discussion

Our exploration into the utilization of GPT models for medical coding reveals several noteworthy challenges. This underscores the importance of carefully crafted prompts to ensure reliable and consistent results. The second challenge is the non-deterministic nature of generative LLMs. Identical prompts applied to different diseases generate stylistically different outputs, and depending on the prompting strategy, these lead to completely hallucinated responses. We included the screenshots of the GPT inaccuracies and hallucinations in the Supplementary Material section. Our findings show some promising results for certain phenotypes but not all. LLMs tend to perform well for phenotypes that are well-documented, particularly those that have clear, standardized definitions and are extensively represented in the training data of these models. Well-documented phenotypes usually have a consistent set of codes, logical criteria, and textual descriptions that LLMs can more easily recognize and replicate. For example, phenotypes such as “Diabetes Mellitus (Type 2), Acute Myocardial Infarction (AMI)” are well-documented with clear clinical criteria and extensive coding in systems like ICD and SNOMED. Table 6 presents the hallucinations of the GPT model. One of the bigger dangers here is the generation of hallucinations when asking for specific coding systems to standardize the definitions. While this could be easily overcome by using knowledge graphs [19], some codes are completely fabricated and will never map to anything, as recently shown by Soroush et al. [20].

**Table 6:** Hallucinations of GPT 3.5 and 4

Model	Vocab	Logic	Code	String
GPT 3.5	ICD-10	Exclusion	Z91.010	Allergy to nuts
GPT 4	ICD-10	Inclusion	Z91.010	History of peanut allergy

## Future work

The next phase of this work will involve actually using the extracted phenotype definitions by the GPT models and comparing the patient cohorts they select. We will use tools available within the OHDSI community, such as Cohort Diagnostics [21] and PheValuator [22], to observe if these definitions are even close to human generated definitions. Our hypothesis here is that since the LLM identifies the most commonly used codes, and logic, for these definitions, it should do a decent job in identifying the core of the phenotype cohort, without including additional edge cases, thus not leaving too many patients out. Success will be measured by the similarity between these cohorts if they closely align; it signifies a significant achievement. Conversely, substantial differences would indicate a need for further refinement of the models, highlighting the iterative nature of improving LLM applications in healthcare. This rigorous evaluation process is essential for advancing our understanding and application of AI in enhancing medical research and patient care.

## Conclusions

Our exploration into utilizing LLMs for automating phenotype definition extraction presents a promising avenue for enhancing the scalability and efficiency of phenotyping in digital healthcare. While our results underscore the potential of LLMs, particularly GPT-3.5 and GPT-4, in generating medically relevant codes, they also highlight the challenges of consistency in textual output and the occurrence of inaccuracies. The critical insight from our study is the importance of developing robust evaluation and validation frameworks to ensure the reliability of LLM outputs. The findings indicate that despite the hallucinations and inconsistencies, GPT models hold potential value as an initial step or augmentation tool in the phenotyping process which could significantly streamline and enhance electronic phenotyping methodologies.

## Data Availability

Data and code is provided at the following repository: https://github.com/jmbanda/blah8.

## Abbreviations

EHRs: Electronic health records
NLP: Natural language processing
LLMs: Large language models
PheKB: Phenotype knowledge base
OHDSI: Observational Health Data Sciences and Informatics
HDRUK: Health Data Research UK
OMOP: Observational Medical Outcomes Partnership
CDM: Common data model
